# Comparison of Safety and Efficacy Between Clopidogrel and Ticagrelor in Elderly Patients With Acute Coronary Syndrome: A Systematic Review and Meta-Analysis

**DOI:** 10.3389/fphar.2021.743259

**Published:** 2021-10-18

**Authors:** Xiangkai Zhao, Jian Zhang, Jialin Guo, Jinxin Wang, Yuhui Pan, Xue Zhao, Wentao Sang, Kehui Yang, Fengyang Xu, Feng Xu, Yuguo Chen

**Affiliations:** ^1^ Department of Emergency Medicine, Qilu Hospital, Shandong University, Jinan, China; ^2^ Chest Pain Center, Qilu Hospital, Shandong University, Jinan, China; ^3^ Shandong Provincial Clinical Research Center for Emergency and Critical Care Medicine, Institute of Emergency and Critical Care Medicine of Shandong University, Qilu Hospital, Shandong University, Jinan, China; ^4^ Key Laboratory of Emergency and Critical Care Medicine of Shandong Province, Key Laboratory of Cardiopulmonary-Cerebral Resuscitation Research of Shandong Province, Shandong Provincial Engineering Laboratory for Emergency and Critical Care Medicine, Qilu Hospital, Shandong University, Jinan, China; ^5^ The Key Laboratory of Cardiovascular Remodeling and Function Research, Chinese Ministry of Education, Chinese Ministry of Health and Chinese Academy of Medical Sciences, The State and Shandong Province Joint Key Laboratory of Translational Cardiovascular Medicine, Qilu Hospital, Shandong University, Jinan, China

**Keywords:** elderly patients, ticagrelor, clopidogrel, acute coronary syndrome, meta-analysis

## Abstract

**Background:** Dual antiplatelet therapy combining aspirin with a P2Y12 adenosine diphosphate receptor inhibitor is a therapeutic mainstay for acute coronary syndrome (ACS). However, the optimal choice of P2Y12 adenosine diphosphate receptor inhibitor in elderly (aged ≥65 years) patients remains controversial. We conducted a meta-analysis to compare the efficacy and safety of ticagrelor and clopidogrel in elderly patients with ACS. **Methods:** We comprehensively searched in Web of Science, EMBASE, PubMed, and Cochrane databases through 29^th^ March, 2021 for eligible randomized controlled trials (RCTs) comparing the efficacy and safety of ticagrelor or clopidogrel plus aspirin in elderly patients with ACS. Four studies were included in the final analysis. A fixed effects model or random effects model was applied to analyze risk ratios (RRs) and hazard ratios (HRs) across studies, and I^2^ to assess heterogeneity.

**Results:** A total number of 4429 elderly patients with ACS were included in this analysis, of whom 2170 (49.0%) patients received aspirin plus ticagrelor and 2259 (51.0%) received aspirin plus clopidogrel. The ticagrelor group showed a significant advantage over the clopidogrel group concerning all-cause mortality (HR 0.78, 95% CI 0.63–0.96, I^2^ = 0%; RR 0.79, 95% CI 0.66–0.95, I^2^ = 0%) and cardiovascular death (HR 0.71, 95% CI 0.56–0.91, I^2^ = 0%; RR 0.76, 95% CI 0.62–0.94, I^2^ = 5%) but owned a higher risk of PLATO major or minor bleeding (HR 1.46, 95% CI 1.13–1.89, I^2^ = 0%; RR 1.40, 95% CI 1.11–1.76, I^2^ = 0%). Both the groups showed no significant difference regarding major adverse cardiovascular events (MACEs) (HR 1.06, 95% CI 0.68–1.65, I^2^ = 77%; RR 1.04, 95% CI 0.69–1.58, I^2^ = 77%).

**Conclusion:** For elderly ACS patients, aspirin plus ticagrelor reduces cardiovascular death and all-cause mortality but increases the risk of bleeding. Herein, aspirin plus ticagrelor may extend lifetime for elderly ACS patients compared with aspirin plus clopidogrel. The optimal DAPT for elderly ACS patients may be a valuable direction for future research studies.

## Introduction

Dual antiplatelet therapy (DAPT) combining aspirin with a P2Y12 inhibitor is recommended in patients with ACS (Roffi et al., 2016; Ibanez et al., 2018; Collet et al., 2021). The common P2Y12 inhibitors contain ticagrelor, clopidogrel, prasugrel, and cangrelor (Valgimigli et al., 2018). Elderly ACS patients are commonly accompanied with a higher risk of recurrent ischemic events as well as bleeding complications, raising a critical challenge of selecting the optimal antiplatelet medicine (Capodanno and Angiolillo, 2010). The current guidelines did not give a clear answer to this question (Valgimigli et al., 2018). The problem of antiplatelet strategies in elderly ACS patients had received more clinical attention in recent years, and high-quality clinical studies have been published successively.

Contemporary data from observational studies and randomized controlled trials (RCTs) showed conflicting reports regarding the preference of ticagrelor or clopidogrel in elderly ACS patients. The results of the POPular AGE trial revealed that among elderly patients with non-ST elevation acute coronary syndrome (NSTE-ACS), clopidogrel reduced bleeding events without increasing the combined endpoints of all-cause mortality, myocardial infarction, stroke, and bleeding (Gimbel et al., 2020). However, the summary from a study about the efficacy and safety outcomes of ticagrelor compared with clopidogrel in elderly Chinese ACS patients showed that ticagrelor reduced the risk of MACEs without increasing the risk of bleeding (Wang and Wang, 2016). Besides, several subgroup data of RCTs drew debatable conclusions, and the analysis from the SWEDEHEART registry showed that aspirin plus ticagrelor was associated with a higher mortality, provoking some uncertainties on its use among the elderly (Wallentin et al., 2009; Husted et al., 2012; Capranzano and Angiolillo, 2020; Szummer et al., 2020). Current research studies could not give a perfect answer to this problem. Herein, we planned to conduct a meta-analysis to compare the efficacy and safety of ticagrelor and clopidogrel in elderly patients with ACS.

## Materials and Methods

### Data Sources and Study Search Strategy

We comprehensively searched in Web of Science, EMBASE, PubMed, and Cochrane databases through 29^th^ March, 2021 for eligible randomized controlled trials (RCTs) using the following keywords: “acute coronary syndrome,” “unstable angina,” “acute myocardial infarction,” “STEMI,” “NSTEMI,” “dual therapy,” “clopidogrel,” and “ticagrelor.”

### Inclusion and Exclusion Criteria

Publications were included if they met the following conditions: 1) the design was a randomized clinical trial, 2) studies contained patients with acute coronary syndrome who were aged ≥65 years, 3) studies compared ticagrelor with clopidogrel, and 4) studies reported all-cause mortality, cardiovascular death, myocardial infarction, stroke, or any bleeding events.

We excluded studies that 1) did not report subgroup results about patients aged ≥65 years and 2) were not designed for humans.

### Data Extraction and Endpoints

Two reviewers (Xiangkai Zhao and Jian Zhang) independently extracted data based on the clinical characteristics of the patients, antiplatelet drugs, inclusion criteria, exclusion criteria, and outcomes after a 1-year follow-up.

The primary endpoints were defined as critical death events (all-cause mortality and cardiovascular death). The secondary endpoints were MACEs (a composite of cardiovascular death, myocardial infarction, or stroke) and bleeding events (PLATO major bleeding, PLATO minor bleeding, fatal bleeding, and PLATO major bleeding or PLATO minor bleeding).

### Methodological Quality

The study selection, data collection, analysis, and reporting of the results were performed on the basis of the Cochrane Handbook for Systematic Reviews of Interventions published by the Cochrane Collaboration. We conducted statistical analysis using the RevMan 5 [Review Manager (RevMan) computer program, version 5.4.1, the Cochrane Collaboration, 2020]. Heterogeneity was assessed by the Q-statistic (*p* ≤ 0.05 was considered statistically significant) and I^2^ statistical test. Publication bias was visually estimated by using funnel plots. The fixed effects model (I^2^ ≤ 50%) or random effects model (I^2^ > 50%) was used to analyze risk ratios (RRs) or hazard ratios (HRs) across studies. Sensitivity analysis was carried out by using the method of checking the influence of an individual trial on the pooled endpoints by excluding each trial solely. Any discrepancies between the reviewers were solved by discussion. Risk of bias composites randomization generation, allocation concealment, blinding assessment, completeness of follow-up, absence of selective reporting, and other potential biases. The risk of bias of each study was assessed by the Cochrane Collaboration tool. The Grading of Recommendation, Assessment, Development, and Evaluation (GRADE) tool was used for the assessment of the reliability of each outcome. The certainty of the evidence was appraised as high, moderate, low, or very low.

## Compliance With Ethics Guidelines

We conducted this study based on previously published studies, and no patients or public were involved in this study.

## Results

### Study Selection

**FIGURE 1:**
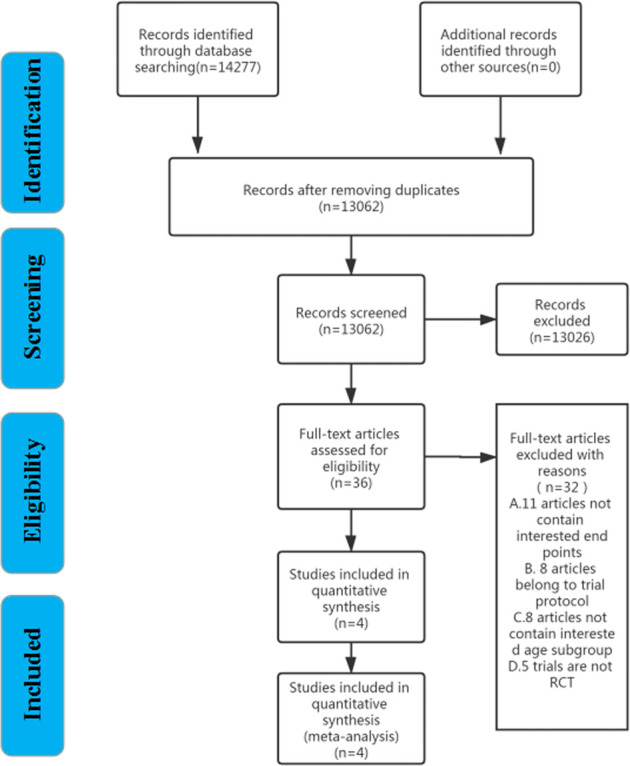
Flow diagram of the literature search and study.

In total, 13,062 articles were identified through the Web of Science, EMBASE, PubMed, and Cochrane databases (Supplementary Table S1). 13,026 articles were excluded by screening the title and abstract. At last, a total of 36 articles in full text were read, and only four clinical trials (Wallentin et al., 2009; Wang and Wang, 2016; Park et al., 2019; Gimbel et al., 2020) fulfilled the eligibility criteria. The literature search and screening processes are presented in Figure 1.

### Major Characteristics of the Included Studies

The characteristics of the included studies are shown in Table 1. Four studies were included in the final analysis, and all four were prospective randomized controlled trials and reported their results for the 1-year follow-up period. A total number of 4,429 elderly patients with ACS were included in this analysis, 2,170 (49.0%) patients received aspirin plus ticagrelor, and 2,259 (51.0%) received aspirin plus clopidogrel. All participants were 65 years or older. ACS patients were treated with ticagrelor or clopidogrel on the basis of aspirin.

**TABLE 1:** Characteristics of the included trials.

	Duk-Woo 2020		Gimbel et al. (2020)		Huidong Wang and Wang (2016)		Wallentin et al. (2009)	
	Ti	Cl	Ti	Cl	Ti	Cl	Ti	Cl
Study type	RCT		RCT		RCT		RCT	
Age (years)	≥65		≥70		≥65		≥75	
Number of patients	172	177	502	500	100	100	1396	1482
Men	ND		65%	63%	69%	66%	56.5%	
Hypertension	ND		73%	73%	79%	82%	75.2%	
Diabetes	ND		30%	29%	42%	39%	28.1%	
Dyslipidemia	ND		65%	65%	84%	79%	46.1%	
Prior MI	ND		27%	24%	17%	15%	26.5%	
Prior PCI	ND		24%	20%	3%	5%	14.6%	
Prior CABG	ND		17%	17%	0	0	8.9%	
CHF	ND		ND	ND	13%	19%	10.5%	
TIA	ND		8%	7%	16%	14%	4.8%	
Smoker	ND		13%	14%	37%	41%	10%	
Diagnosis								
STEMI	ND		0	0	37%	32%	25.9%	
NSTEMI	ND		86%	86%	44%	47%	52.6%	
Unstable angina	ND		11%	11%	19%	21%	19.1%	
Coronary angiography during study	ND		90%	88%	86%	83%	44%	
PCI during study	ND		48%	46%	75%	71%	23.2%	
CABG during study	ND		17%	16%	0	0	8.1%	
Drug dose								
LD	180 mg.bid	600 mg.qd	180 mg.bid	300 or 600 mg.qd	180 mg.bid	300 mg.qd	180 mg.bid	300–600 mg.qd
MD	90 mg.bid	75 mg.qd	90 mg.bid	75 mg.qd	90 mg.bid	75 mg.qd	90 mg.bid	75 mg.qd
Follow-up (month)	12		12		12		12	
Clinical events	MACEs and PLATO major or minor bleeding		MACEs and PLATO major and minor bleeding,12,3,4,5,6,7,8		MACEs, 1,2,3,4,5,6, and 7		MACEs,1,2,3,4, and 8	

Ti, ticagrelor; Cl, clopidogrel; LD, loading dose; MD, maintenance dose; MACEs, major adverse cardiovascular events; MI, myocardial infarction; PCI, percutaneous coronary intervention; CABG, coronary artery bypass grafting; CHF, chronic cardiac failure; TIA, transient ischemic attack; STEMI, ST segment elevation myocardial infarction; NSTEMI, non–ST-elevation myocardial infarction; ND, no data; qd, once a day; bid, twice a day. 1 = all-cause mortality, 2 = cardiovascular death, 3 = MI, 4 = stroke, 5 = PLATO major bleeding, 6 = plato minor bleeding, 7 = life-threatening bleeding, 8 = stent thrombosis.

### Risk of Bias and Study Quality

The assessment of the risk of bias of each study is shown in Supplementary Figure S1, and all four trials had shown a low risk of bias. GRADE assessment shows that the outcomes of MACEs, all-cause mortality, cardiovascular death, MI, and stroke are of high quality, and PLATO major bleeding, PLATO minor bleeding, fatal bleeding, PLATO major or minor bleeding, and stent thrombosis are of low quality (Supplementary Table S2). We used the Preferred Reporting Items for Systematic Reviews and Meta-Analyses (PRISMA) checklist to improve the reporting quality of our study (Supplementary Table S3).

### Primary Outcomes

**FIGURE 2:**
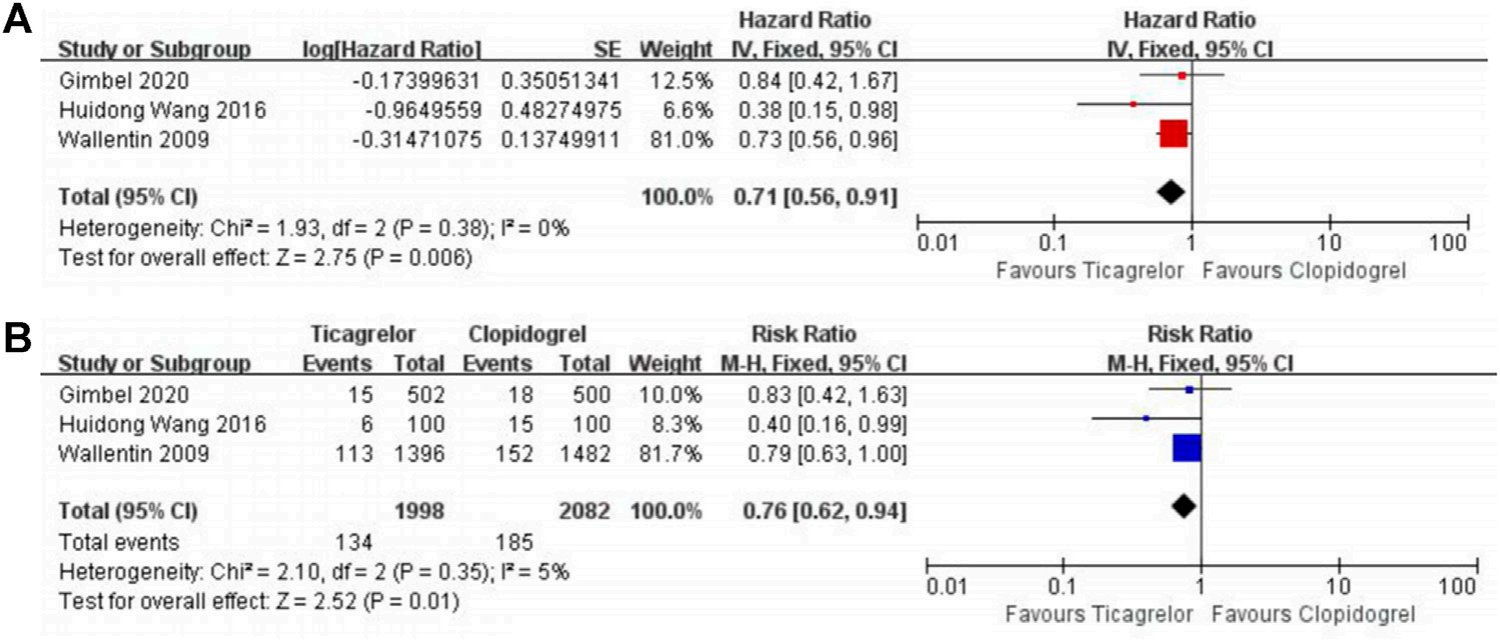
Meta-analysis with HR **(A),** RR **(B)**, and 95% CI for all-cause mortality. Boxes are the relative risk estimates from each study; the horizontal bars are 95% CI. The size of the box is proportional to the weight of the study in the meta-analysis. HR, hazard ratio; RR, risk ratio; CI, confidence interval.

**FIGURE 3:**
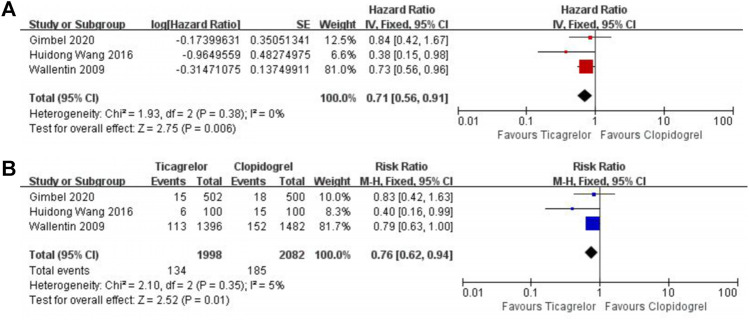
Meta-analysis with HR **(A),** RR **(B)**, and 95% CI for cardiovascular death. Boxes are the relative risk estimates from each study; the horizontal bars are 95% CI. The size of the box is proportional to the weight of the study in the meta-analysis. HR, hazard ratio; RR, risk ratio; CI, confidence interval.

After a 1-year follow-up, elderly ACS patients who received ticagrelor performed a lower event rate in all-cause mortality (HR 0.78, 95% CI 0.63–0.96, I^2^ = 0%; RR 0.79, 95% CI 0.66–0.95, I^2^ = 0%) (Figure 2) and cardiovascular death (HR 0.71, 95% CI 0.56–0.91, I^2^ = 0%; RR 0.76, 95% CI 0.62–0.94, I^2^ = 5%) (Figure 3).

### Secondary Outcomes

**FIGURE 4:**
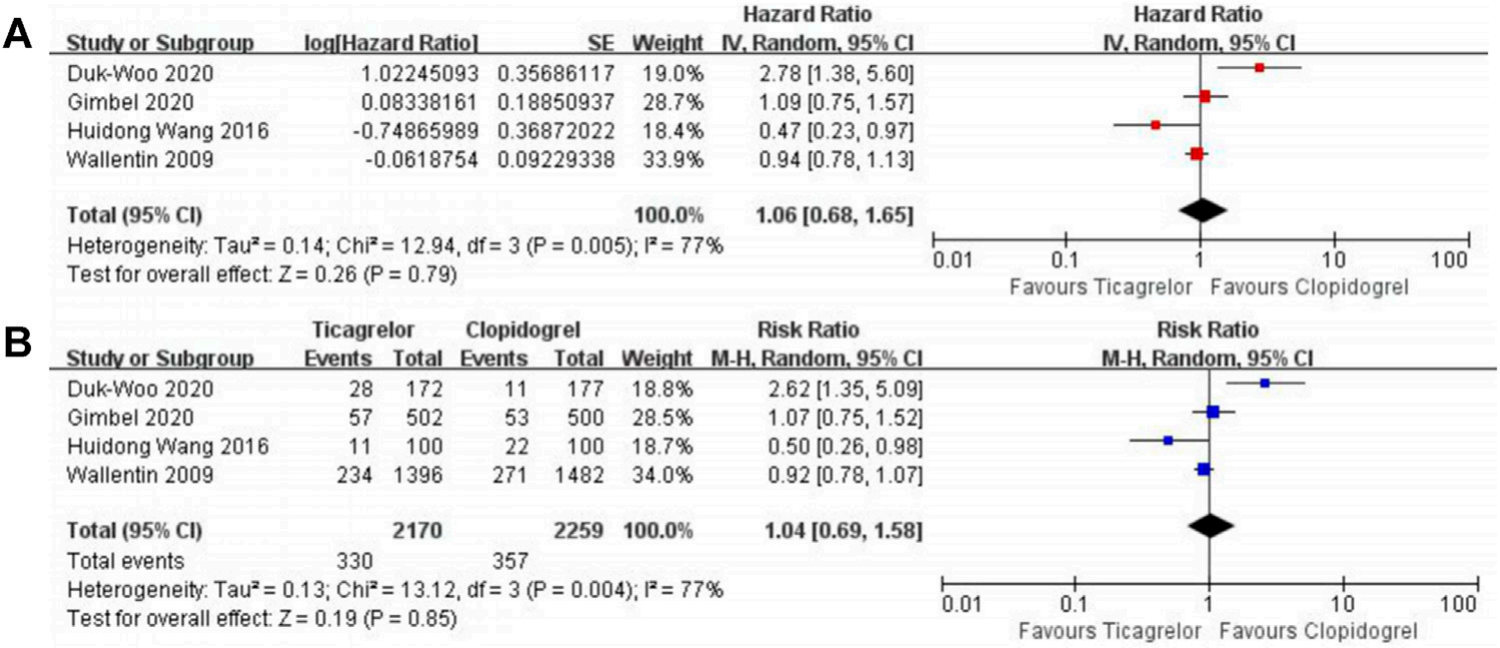
Meta-analysis with HR **(A),** RR **(B)**, and 95% CI for the composite of cardiovascular death, myocardial infarction, and stroke. The boxes are the relative risk estimates from each study; the horizontal bars are 95% CI. The size of the box is proportional to the weight of the study in the meta-analysis. HR, hazard ratio; RR, risk ratio; CI, confidence interval.

**FIGURE 5:**
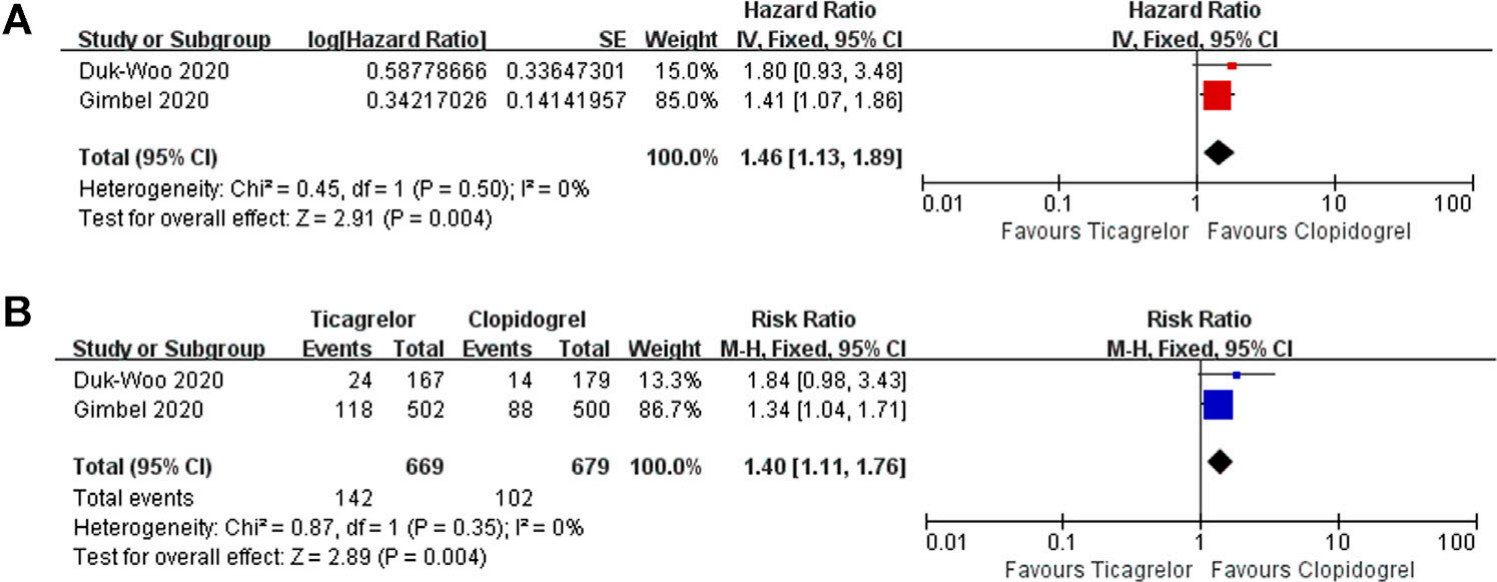
Meta-analysis with HR **(A),** RR **(B)**, and 95% CI for the PLATO major bleeding or minor bleeding. Boxes are the relative risk estimates from each study; the horizontal bars are 95% CI. The size of the box is proportional to the weight of the study in the meta-analysis. HR, hazard ratio; RR, risk ratio; CI, confidence interval.

**FIGURE 6:**
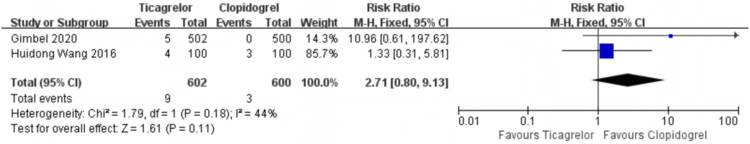
Meta-analysis with RR and 95% CI for fatal bleeding. Boxes are the relative risk estimates from each study; the horizontal bars are 95% CI. The size of the box is proportional to the weight of the study in the meta-analysis. HR, hazard ratio; RR, risk ratio; CI, confidence interval.

After 1 year of receiving DAPT therapy, ticagrelor showed clinical equipoise in terms of MACEs (HR 1.06, 95% CI 0.68–1.65, I^2^ = 77%; RR 1.04, 95% CI 0.69–1.58, I^2^ = 77%) (Figure 4), MI (HR 0.92, 95% CI 0.74–1.15, I^2^ = 44%; RR 0.94, 95% CI 0.77–1.14, I^2^ = 45%) (Supplementary Figure S2), stroke (HR 1.55,95% CI 0.98–2.46, I^2^ = 0%; RR 2.05, 95% CI 1.31–3.22, I^2^ = 0%) (Supplementary Figure S3), and stent thrombosis (RR 0.44, 95% CI 0.06–3.42, I^2^ = 57%) (Supplementary Figure S4). As for bleeding risk, the patients who received ticagrelor showed a higher risk of PLATO major or minor bleeding (HR 1.46, 95% CI 1.13–1.89, I^2^ = 0%; RR 1.40, 95% CI 1.11–1.76, I^2^ = 0%) (Figure 5). When focused on lethal bleeding (RR 2.71, 95% CI 0.8–9.13, I^2^ = 44%) (Figure 6), both PLATO major bleeding (HR 1.39, 95% CI 0.94–2.04, I^2^ = 0%; RR 1.38, 95% CI 0.95–2.00, I^2^ = 0%) (Supplementary Figure S5) and PLATO minor bleeding (HR 1.37, 95% CI 1.00–1.90, I^2^ = 0%; RR 1.33, 95% CI 0.99–1.80, I^2^ = 0%) (Supplementary Figure S6) groups showed a similar risk.

## Discussion

The primary finding of our study is that after a 1-year follow-up, aspirin plus ticagrelor reduced all-cause mortality and cardiovascular death but owned a higher risk of PLATO major or minor bleeding than aspirin plus clopidogrel. Our findings provided a new clinical thought for a better choice of P2Y12 adenosine diphosphate receptor inhibitor for elderly ACS patients.

Dual antiplatelet therapy combining aspirin with a P2Y12 adenosine diphosphate receptor inhibitor is a standard regimen for ACS. For the included RCTs, all studies were conducted on the basis of aspirin, so the end events were used for comparing the safety and efficacy between clopidogrel and ticagrelor.

The current guidelines recommended aspirin plus ticagrelor in patients with acute coronary syndrome (Valgimigli et al., 2018), but there was no explicit suggestion for elderly ACS patients. According to the report of the Philippine Heart Association ACS registry, the incidence of ACS was higher in the elderly (Reano et al., 2020). At the same time, older age groups were often excluded from clinical studies. Thus, the optimal choice of P2Y12 adenosine diphosphate receptor inhibitor for elderly patients was necessary to be confirmed as soon as possible. Data from different studies did not reach a consensus for this academic problem. In the famed PLATO study, the ticagrelor group showed significant advantages in reducing the risk of cardiovascular death (HR 0.73, CI 0.56–0.96, *p* = 0.47) and all-cause mortality (HR 0.77, CI 0.60–0.98, *p* = 0.76) *versus* clopidogrel in the subgroup which contained patients aged ≥75 years old, without increasing the overall bleeding risk (HR 1.02, CI 0.82–1.27, *p* = 0.89) (Wallentin et al., 2009). However, an RCT aiming to explore efficacy and safety outcomes of ticagrelor compared with clopidogrel in elderly Chinese ACS patients drew a different conclusion. Ticagrelor owned an extra advantage in reducing the risk of a composite of cardiovascular death, MI, and stroke (HR 0.473, CI 0.230–0.976, *p* = 0.043) (Wang and Wang, 2016). Surprisingly, the results of the POPular AGE trial revealed that ticagrelor increased the risk of bleeding with no superior co-primary net clinical benefit outcome (Gimbel et al., 2020), which posed a challenge to the traditional opinion about the ticagrelor advantage theory and stimulated our interests in exploring this issue. Our research studies supported the ticagrelor advantage theory that ticagrelor could reduce the risk of all-cause mortality and cardiovascular death. In other words, ticagrelor may extend survival time for elderly ACS patients compared with clopidogrel.

For the outcomes of MACEs, ticagrelor and clopidogrel showed comparable clinical benefits. The probable sources of heterogeneity for the outcomes of MACEs may come from different age cutoffs (Wallentin et al., 2009). The study by Wallentin et al. (2009) had cutoffs of 65 years (HR) and 75 years (RR), and the remaining study data had cutoffs of 65 (Wang and Wang, 2016), 70 (Gimbel et al., 2020), and 75 (Park et al., 2019) years. According to our results, when focusing on all-cause mortality and cardiovascular death solely, ticagrelor showed a significant advantage compared to clopidogrel in elderly ACS patients. Our results suggested that ticagrelor may have a higher application value in the advanced age-group.

Shreds of evidence suggested that ticagrelor had a higher risk of bleeding (Gimbel et al., 2020; Johnston et al., 2020; Silvain et al., 2020). Our research studies further confirmed this issue. Ages of the patients and drug properties were crucial reasons for higher bleeding risk. Age was a validated predictor of adverse prognosis, and the risk of bleeding increased with age (Eagle et al., 2004). Elderly patients with ACS often have multiple comorbidities, as well as a gradual decline in organismal function with advancing age. Age-related changes in thrombotic status, decreased vascular repair capacity, and clinical factors may lead to a greater difference in the safety of antiplatelet agents in the elderly than in young patients (Lopes and Alexander, 2009). As for the medication itself, ticagrelor works faster and more effectively. Ticagrelor has a binding site different from adenosine diphosphate, making its inhibition reversible. Besides, it can activate *CYP2C19* without the liver, so ticagrelor has a stronger antiplatelet effect than clopidogrel (Birkeland et al., 2010; Guan et al., 2018). Stronger antiplatelet effect is commonly associated with a higher risk of bleeding, so an appropriate antiplatelet strategy is important for ACS patients, especially for elderly patients.

A higher risk of bleeding is not an absolute contraindication to the application of ticagrelor. According to our results, ticagrelor is a preferable choice compared with clopidogrel in reducing the occurrence of death in most elderly ACS patients. The occurrence of fatal or irreversible bleeding events is one of the main factors affecting the long-term survival of patients. Our results revealed that ticagrelor or clopidogrel shared a similar risk of fatal bleeding. Additional management measures were necessary for patients with ACS who were under a higher bleeding risk. For this issue, a recent clinical investigation indicated that ticagrelor monotherapy might be a suitable alternative option (Mehran et al., 2019). In addition, shortening DAPT duration (Palmerini et al., 2017) and enhancing the education of high-risk patients and their families are valuable in preventing bleeding events. Fatal or irreversible ischemia was also a clinically significant event for elderly ACS patients, the ATLAS ACS 2-TIMI 51 revealed an interesting phenomenon, and rivaroxaban therapy at an oral dose of 2.5 mg twice daily in patients treated with aspirin and clopidogrel was associated with a net reduction in fatal or irreversible events (Gibson et al., 2018). Therefore, with reasonable precautions, the risk of bleeding with ticagrelor could be minimized as much as possible.

Our meta-analysis has several advantages over previous research studies. First, we were the first to systematically conduct a meta-analysis of clopidogrel *versus* ticagrelor in elderly patients with ACS. Second, we used both RR and HR to comprehensively demonstrate the findings. Third, for high bleeding risk, we conducted a systematic analysis and gave reasonable monitoring and preventive suggestions. Finally, this meta-analysis gave a new direction for the prospective studies.

There are several limitations to our meta-analysis. First, our meta-analysis only contains four RCTs which may not reflect the real world, and two datasets of the included articles come from the subgroup analysis. We could not get individual patient data, so a detailed age-stratified analysis or the analysis of other bleeding definitions could not be performed. Second, in the study of the POPular AGE trial, 5% of patients in the ticagrelor group received prasugrel, which may result in the final results being subjected to some errors. Third, two studies are open-labeled randomized trials, introducing the potential for latent performance bias. In addition, we found the heterogeneity of Duk-Woo 2020 was significant when the heterogeneity tests were performed by sequential deletion. The probable reason might be that most patients received percutaneous coronary intervention in Duk-Woo 2020.

## Conclusion

Our results reveal that aspirin plus ticagrelor reduces cardiovascular death and all-cause mortality but increases the risk of PLATO major bleeding or PLATO minor bleeding when compared with aspirin plus clopidogrel in elderly ACS patients. Herein, aspirin plus ticagrelor may extend lifetime for elderly ACS patients compared with aspirin plus clopidogrel. The low sample size of current studies cannot support a definite conclusion for this vital issue. Further studies focusing on DAPT of elderly ACS patients with larger population are still needed.

## Data Availability

The original contributions presented in the study are included in the article/Supplementary Material; further inquiries can be directed to the corresponding authors.
