# Supplementary material for: Comparison of Safety and Efficacy Between Clopidogrel and Ticagrelor in Elderly Patients With Acute Coronary Syndrome: A Systematic Review and Meta-Analysis
Source: Front Pharmacol. 2021 Oct 18;12:743259. doi: 10.3389/fphar.2021.743259 (PMC8552409; doi:10.3389/fphar.2021.743259)
Supplement: Supplementary file 1 [file DataSheet1.ZIP › Supplementary files/Supplementary_Material.docx]

Supplementary Material

# Supplementary Figures and Tables

## Supplementary Figures


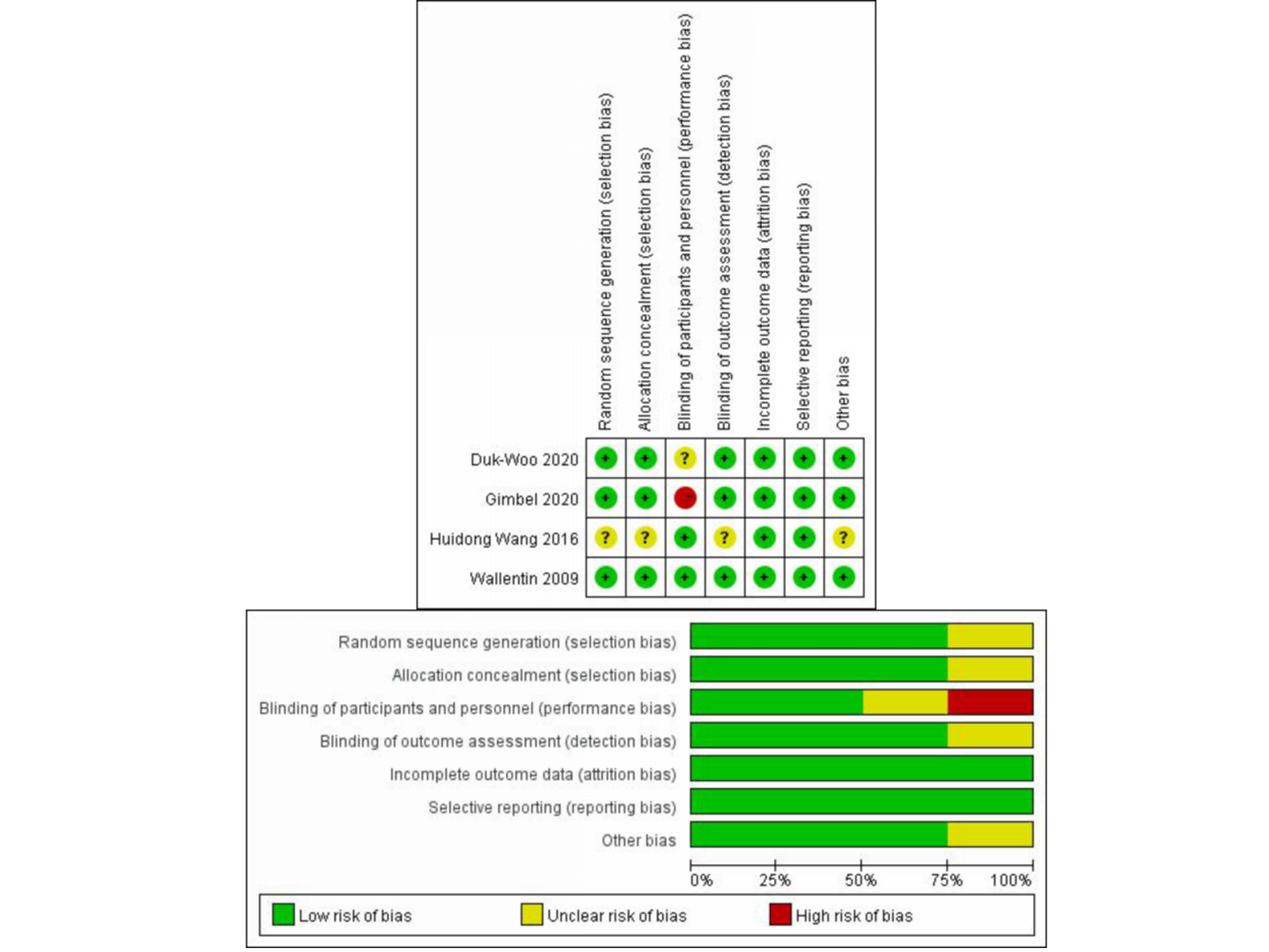


**Supplementary Figure 1.** Risk of bias of each study


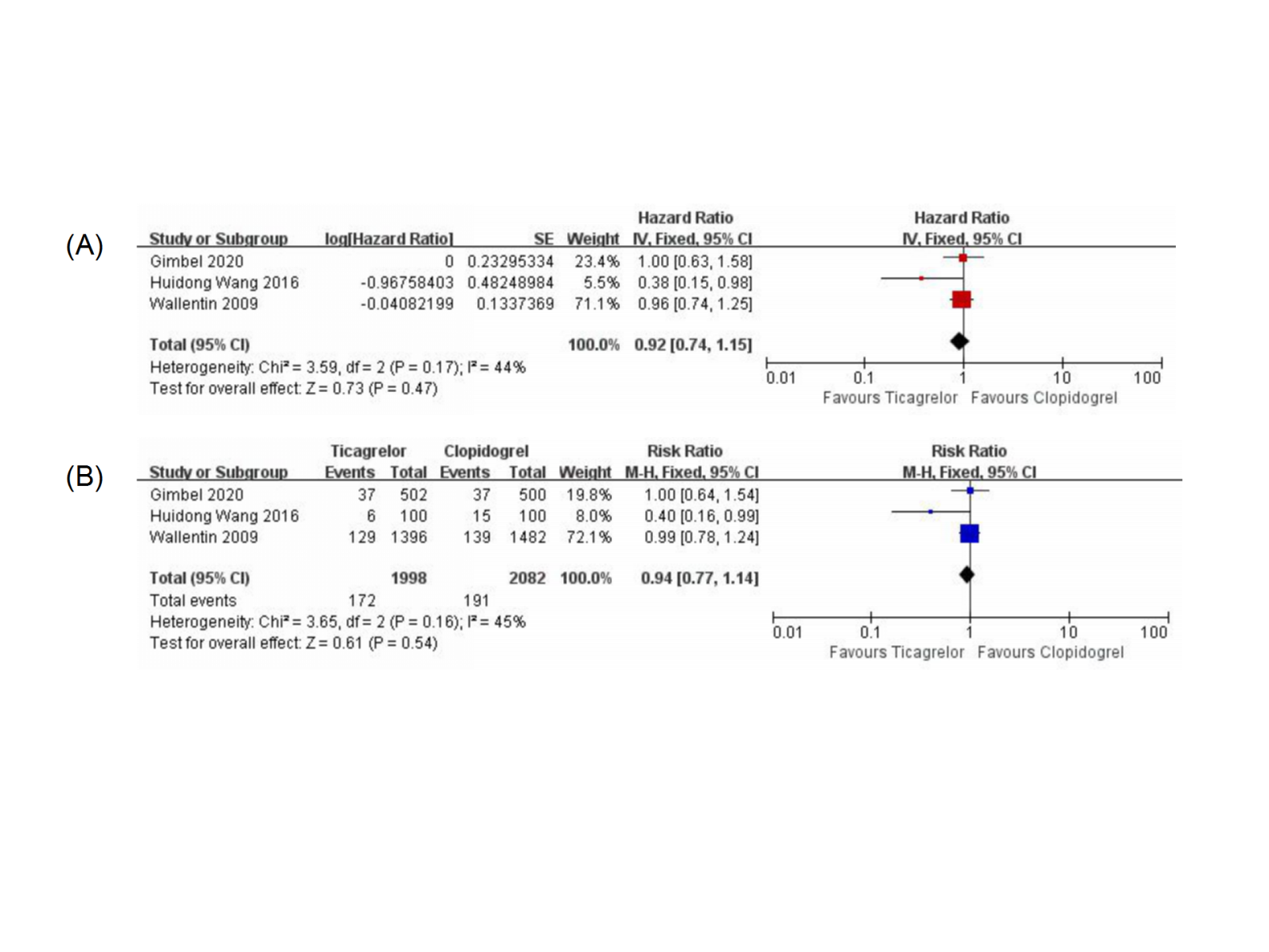


**Supplementary Figure 2.** Meta-analysis with HR (A) and RR (B) and 95% CI for myocardial infarction. Boxes are the relative risk estimates from each study; the horizontal bars are 95% CI. The size of the box is proportional to the weight of the study in the meta-analysis. HR, hazard ratio; RR, risk ratio; CI, confidence interval.


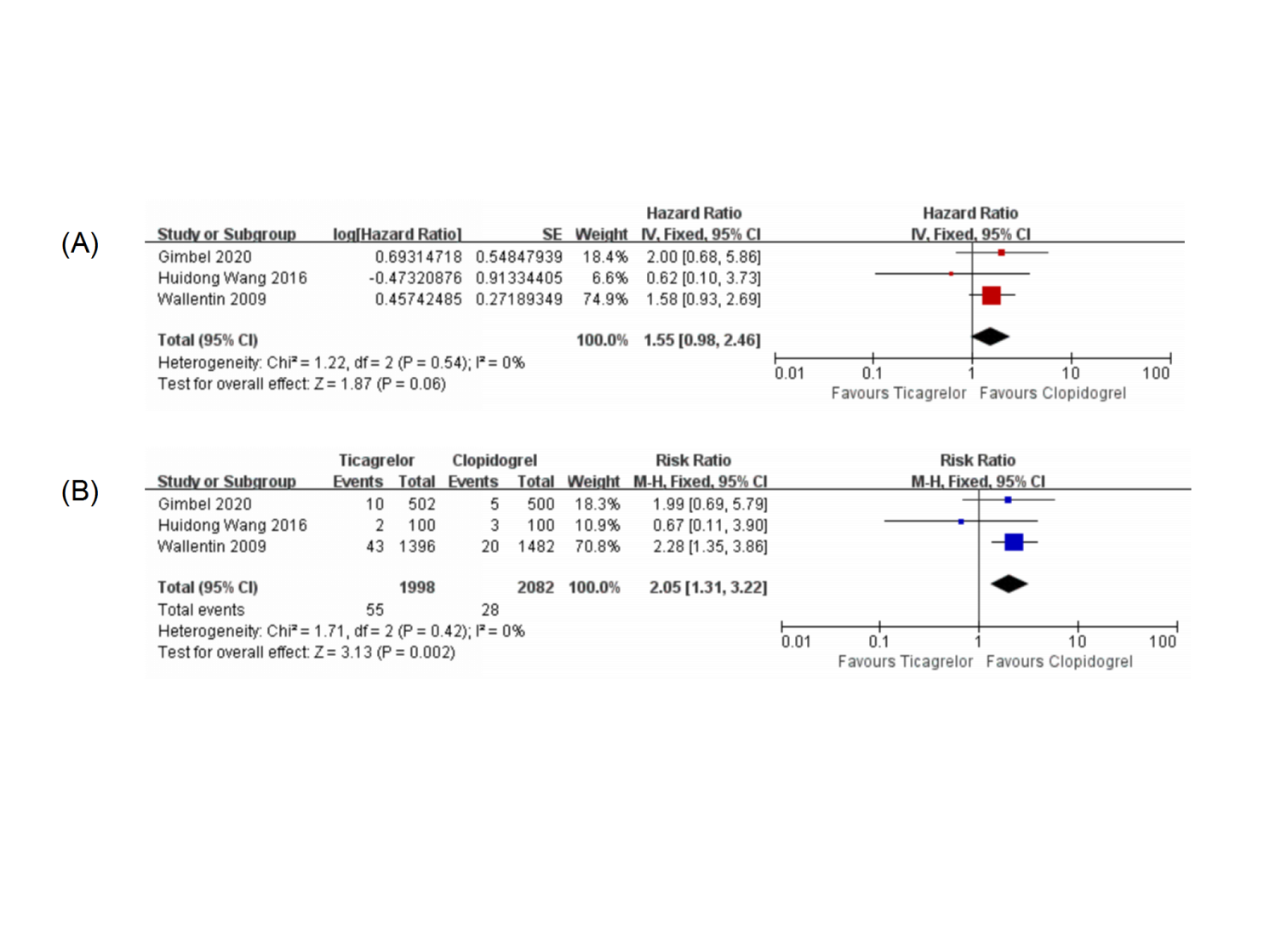


**Supplementary Figure 3.** Meta-analysis with HR (A) and RR (B) and 95% CI for stroke. Boxes are the relative risk estimates from each study; the horizontal bars are 95% CI. The size of the box is proportional to the weight of the study in the meta-analysis. HR, hazard ratio; RR, risk ratio; CI, confidence interval.

**
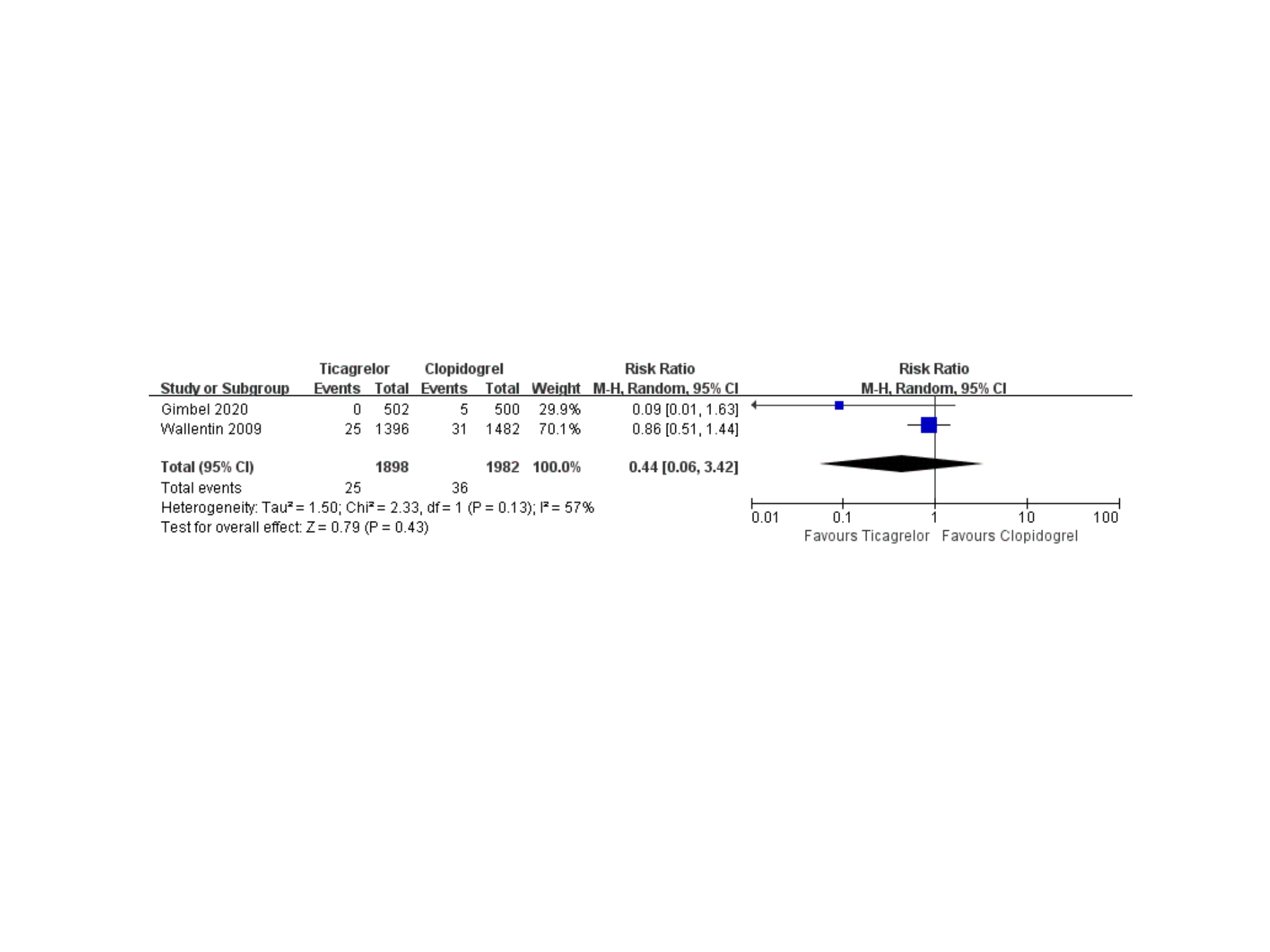
**

**Supplementary Figure 4.** Meta-analysis with RR and 95% CI for stent thrombosis. Boxes are the relative risk estimates from each study; the horizontal bars are 95% CI. The size of the box is proportional to the weight of the study in the meta-analysis. HR, hazard ratio; RR, risk ratio; CI, confidence interval.


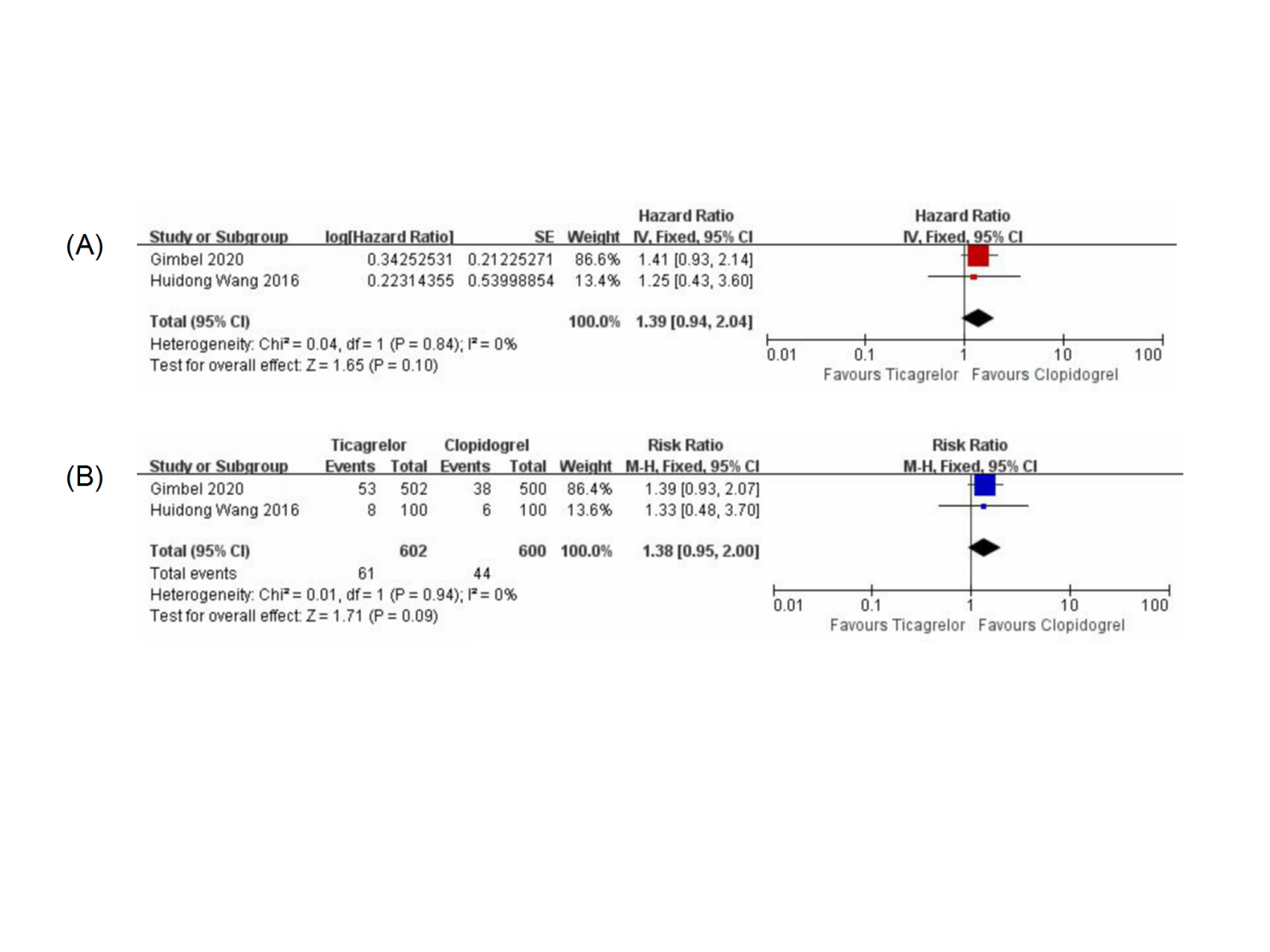


**Supplementary Figure 5.** Meta-analysis with HR (A) and RR (B) and 95% CI for Plato Major bleeding. Boxes are the relative risk estimates from each study; the horizontal bars are 95% CI. The size of the box is proportional to the weight of the study in the meta-analysis. HR, hazard ratio; RR, risk ratio; CI, confidence interval.


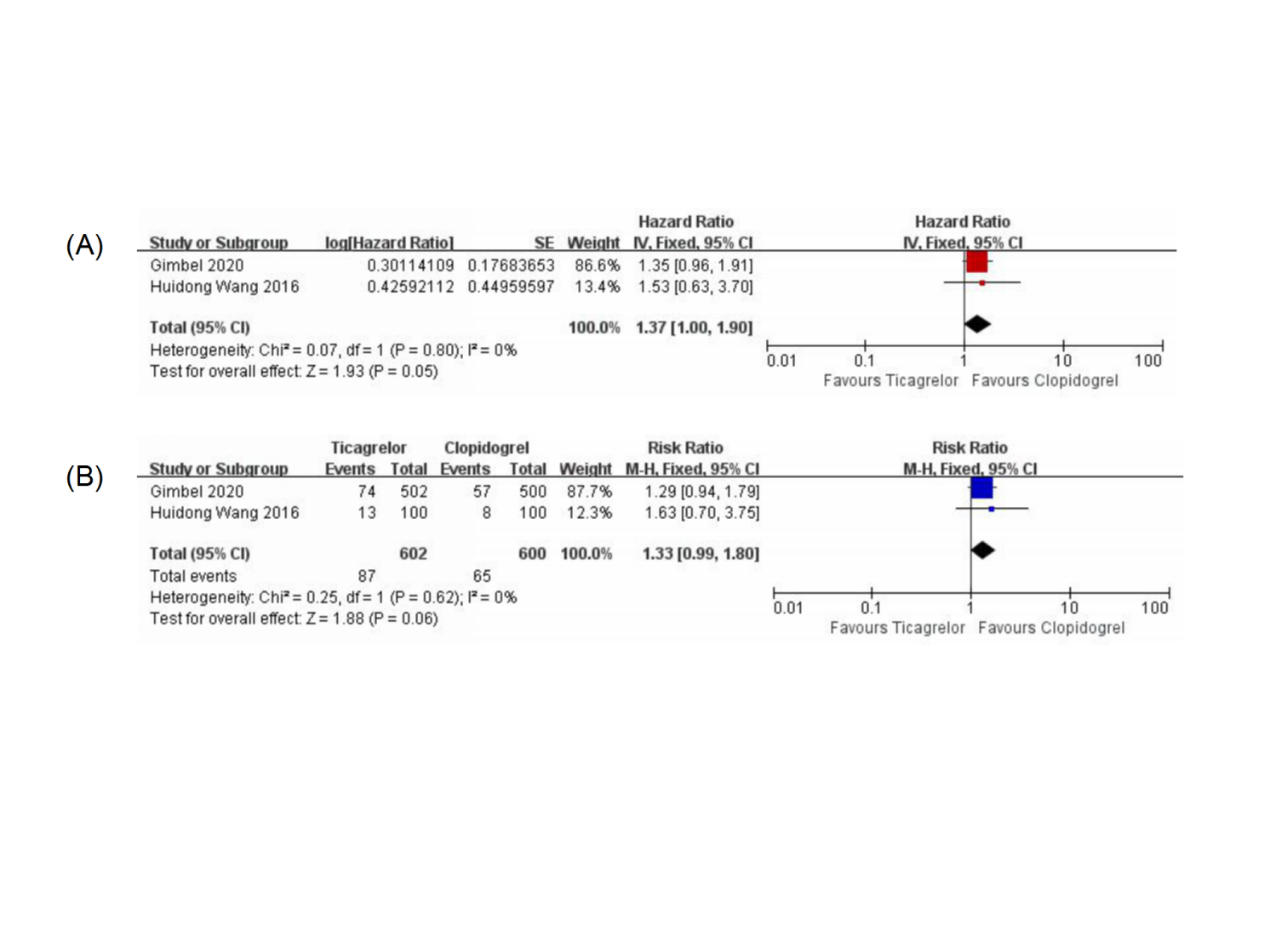


**Supplementary Figure 6.** Meta-analysis with HR (A) and RR (B) and 95% CI for Plato minor bleeding. Boxes are the relative risk estimates from each study; the horizontal bars are 95% CI. The size of the box is proportional to the weight of the study in the meta-analysis. HR, hazard ratio; RR, risk ratio; CI, confidence interval.

# 1.2 Supplementary Tables

| **Database** | **Key words** | **Results (n)** |
| --- | --- | --- |
| PubMed | **(((acute coronary syndrome)[Title/Abstract] OR ACS[Title/Abstract] OR (unstable angina)[Title/Abstract] OR UA[Title/Abstract] OR NSTEMI[Title/Abstract] OR (non-st-segment elevation myocardial infarction)[Title/Abstract] OR NSTEMI[Title/Abstract] OR STEMI[Title/Abstract] OR (st-element myocardial infarction)[Title/Abstract] OR (post PCI)[Title/Abstract] OR (after PCI))[Title/Abstract]) AND ((Clopidogrel[Title/Abstract] OR Ticagrelor[Title/Abstract] OR anticoagulation[Title/Abstract] OR risk[Title/Abstract] OR (prevention of thromboembolic event)[Title/Abstract] OR anti thrombosis[Title/Abstract] OR antithrombotic[Title/Abstract] OR (dual therapy)[Title/Abstract] OR bleeding[Title/Abstract] OR aspirin[Title/Abstract] OR (Dual antiplatelet therapy))[Title/Abstract])** | 8930 |
| EMBASE | clopidogrel:ab,ti AND ticagrelor:ab,ti | 3435 |
| Web of Science | TI=clopidogrel and TI=TICAGRELOR | 669 |
| Cochrane Library | clopidogrel [tiab] AND ticagrelor [tiab] | 1162 |

#

**Supplemental Table 1. Search strategy and results**

| **Quality assessment** | | | | | | | **No of patients** | | **Effect** | | **Quality** |
| --- | --- | --- | --- | --- | --- | --- | --- | --- | --- | --- | --- |
| **No of studies** | **Design** | **Risk of bias** | **Inconsistency** | **Indirectness** | **Imprecision** | **Other considerations** | **RR** | **Control** | **Relative (95% CI)** | **Absolute** |  |
| **MACEs (follow-up 1 years)** | | | | | | | | | | | |
| 4 | randomised trials | no serious risk of bias^1^ | no serious inconsistency | no serious indirectness | no serious imprecision | none | 330/2170  (15.2%) | 357/2259  (15.8%) | RR 1.04 (0.69 to 1.58) | 6 more per 1000 (from 49 fewer to 92 more) | ⊕⊕⊕⊕ HIGH |
|  |  |  |  |  |  |  |  | 14.4% |  | 6 more per 1000 (from 45 fewer to 84 more) |  |
| **All-cause mortality (follow-up 1 years)** | | | | | | | | | | | |
| 3 | randomised trials | no serious risk of bias | no serious inconsistency | no serious indirectness | no serious imprecision | none | 179/1998  (9%) | 236/2082  (11.3%) | RR 0.79 (0.66 to 0.95) | 24 fewer per 1000 (from 6 fewer to 39 fewer) | ⊕⊕⊕⊕ HIGH |
|  |  |  |  |  |  |  |  | 12.4% |  | 26 fewer per 1000 (from 6 fewer to 42 fewer) |  |
| **Cardiovascular death (follow-up 1 years)** | | | | | | | | | | | |
| 3 | randomised trials | no serious risk of bias | no serious inconsistency | no serious indirectness | no serious imprecision | none | 134/1998  (6.7%) | 185/2082  (8.9%) | RR 0.76 (0.62 to 0.94) | 21 fewer per 1000 (from 5 fewer to 34 fewer) | ⊕⊕⊕⊕ HIGH |
|  |  |  |  |  |  |  |  | 10.3% |  | 25 fewer per 1000 (from 6 fewer to 39 fewer) |  |
| **MI (follow-up 1 years)** | | | | | | | | | | | |
| 3 | randomised trials | no serious risk of bias | no serious inconsistency | no serious indirectness | no serious imprecision | none^1^ | 172/1998  (8.6%) | 191/2082  (9.2%) | RR 0.94 (0.77 to 1.14) | 6 fewer per 1000 (from 21 fewer to 13 more) | ⊕⊕⊕⊕ HIGH |
|  |  |  |  |  |  |  |  | 9.4% |  | 6 fewer per 1000 (from 22 fewer to 13 more) |  |
| **Stroke (follow-up 1 years)** | | | | | | | | | | | |
| 3 | randomised trials | no serious risk of bias | no serious inconsistency | no serious indirectness | no serious imprecision | none | 55/1998  (2.8%) | 28/2082  (1.3%) | RR 2.05 (1.31 to 3.22) | 14 more per 1000 (from 4 more to 30 more) | ⊕⊕⊕⊕ HIGH |
|  |  |  |  |  |  |  |  | 1.4% |  | 15 more per 1000 (from 4 more to 31 more) |  |
| **PLATO major bleeding (follow-up 1 years)** | | | | | | | | | | | |
| 2 | randomised trials | serious | no serious inconsistency | no serious indirectness | serious | none | 61/602  (10.1%) | 44/600  (7.3%) | RR 1.38 (0.95 to 2) | 28 more per 1000 (from 4 fewer to 73 more) | ⊕⊕OO LOW |
|  |  |  |  |  |  |  |  | 6.8% |  | 26 more per 1000 (from 3 fewer to 68 more) |  |
| **PLATO minor bleeding (follow-up 1 years)** | | | | | | | | | | | |
| 2 | randomised trials | serious | no serious inconsistency | no serious indirectness | serious | none | 87/602  (14.5%) | 65/600  (10.8%) | RR 1.33 (0.99 to 1.8) | 36 more per 1000 (from 1 fewer to 87 more) | ⊕⊕OO LOW |
|  |  |  |  |  |  |  |  | 9.7% |  | 32 more per 1000 (from 1 fewer to 78 more) |  |
| **fata bleeding (follow-up 1 years)** | | | | | | | | | | | |
| 2 | randomised trials | serious | no serious inconsistency | no serious indirectness | serious | none | 9/602  (1.5%) | 3/600  (0.5%) | RR 2.71 (0.8 to 9.13) | 9 more per 1000 (from 1 fewer to 41 more) | ⊕⊕OO LOW |
|  |  |  |  |  |  |  |  | 1.5% |  | 26 more per 1000 (from 3 fewer to 122 more) |  |
| **PLATO major or minor bleeding (follow-up 1 years)** | | | | | | | | | | | |
| 2 | randomised trials | serious | no serious inconsistency | no serious indirectness | serious | none | 142/669  (21.2%) | 102/679  (15%) | RR 1.4 (1.11 to 1.76) | 60 more per 1000 (from 17 more to 114 more) | ⊕⊕OO LOW |
|  |  |  |  |  |  |  |  | 12.7% |  | 51 more per 1000 (from 14 more to 97 more) |  |
| **stent thrombosis (follow-up 1 years)** | | | | | | | | | | | |
| 2 | randomised trials | serious | serious | no serious indirectness | serious | none | 25/1898  (1.3%) | 36/1982  (1.8%) | RR 0.44 (0.06 to 3.42) | 10 fewer per 1000 (from 17 fewer to 44 more) | ⊕OOO VERY LOW |
|  |  |  |  |  |  |  |  | 1.6% |  | 9 fewer per 1000 (from 15 fewer to 39 more) |  |

**Supplemental Table 2. Quality of outcomes according to** **GRADE**

|  | **#** | **Checklist item** | **Reported on page #** |
| --- | --- | --- | --- |
| **TITLE** | | |  |
| Title | 1 | Identify the report as a systematic review, meta-analysis, or both. | 1 |
| **ABSTRACT** | | |  |
| Structured summary | 2 | Provide a structured summary including, as applicable: background; objectives; data sources; study eligibility criteria, participants, and interventions; study appraisal and synthesis methods; results; limitations; conclusions and implications of key findings; systematic review registration number. | 2 |
| **INTRODUCTION** | | |  |
| Rationale | 3 | Describe the rationale for the review in the context of what is already known. | 2-3 |
| Objectives | 4 | Provide an explicit statement of questions being addressed with reference to participants, interventions, comparisons, outcomes, and study design (PICOS). | 2-3 |
| **METHODS** | | |  |
| Protocol and registration | 5 | Indicate if a review protocol exists, if and where it can be accessed (e.g., Web address), and, if available, provide registration information including registration number. | NA |
| Eligibility criteria | 6 | Specify study characteristics (e.g., PICOS, length of follow-up) and report characteristics (e.g., years considered, language, publication status) used as criteria for eligibility, giving rationale. | 3 |
| Information sources | 7 | Describe all information sources (e.g., databases with dates of coverage, contact with study authors to identify additional studies) in the search and date last searched. | 3 |
| Search | 8 | Present full electronic search strategy for at least one database, including any limits used, such that it could be repeated. | 3 |
| Study selection | 9 | State the process for selecting studies (i.e., screening, eligibility, included in systematic review, and, if applicable, included in the meta-analysis). | 3-4 |
| Data collection process | 10 | Describe method of data extraction from reports (e.g., piloted forms, independently, in duplicate) and any processes for obtaining and confirming data from investigators. | 3-4 |
| Data items | 11 | List and define all variables for which data were sought (e.g., PICOS, funding sources) and any assumptions and simplifications made. | 3 |
| Risk of bias in individual studies | 12 | Describe methods used for assessing risk of bias of individual studies (including specification of whether this was done at the study or outcome level), and how this information is to be used in any data synthesis. | 3-4 |
| Summary measures | 13 | State the principal summary measures (e.g., risk ratio, difference in means). | 3-4 |
| Synthesis of results | 14 | Describe the methods of handling data and combining results of studies, if done, including measures of consistency (e.g., I^2^) for each meta-analysis. | 4-5 |

**Supplemental Table 3.** **PRISMA checklist**
