# Supplementary figures and images for: Comparison of Safety and Efficacy Between Clopidogrel and Ticagrelor in Elderly Patients With Acute Coronary Syndrome: A Systematic Review and Meta-Analysis
Source: Front Pharmacol. 2021 Oct 18;12:743259. doi: 10.3389/fphar.2021.743259 (PMC8552409; doi:10.3389/fphar.2021.743259)

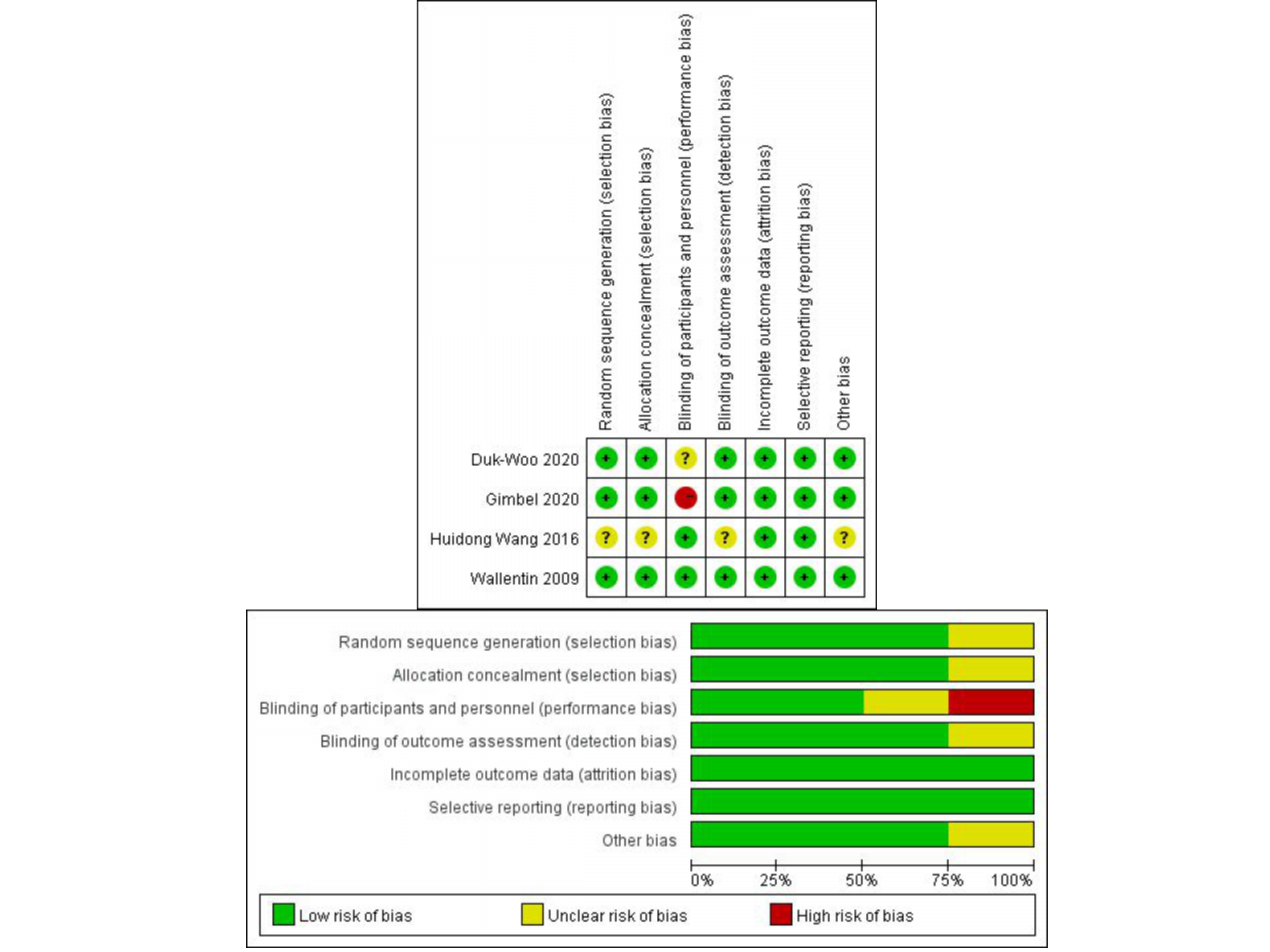

Supplement: Supplementary file 1 [file DataSheet1.ZIP › Supplementary files/Supplementary Figure 1.tif]

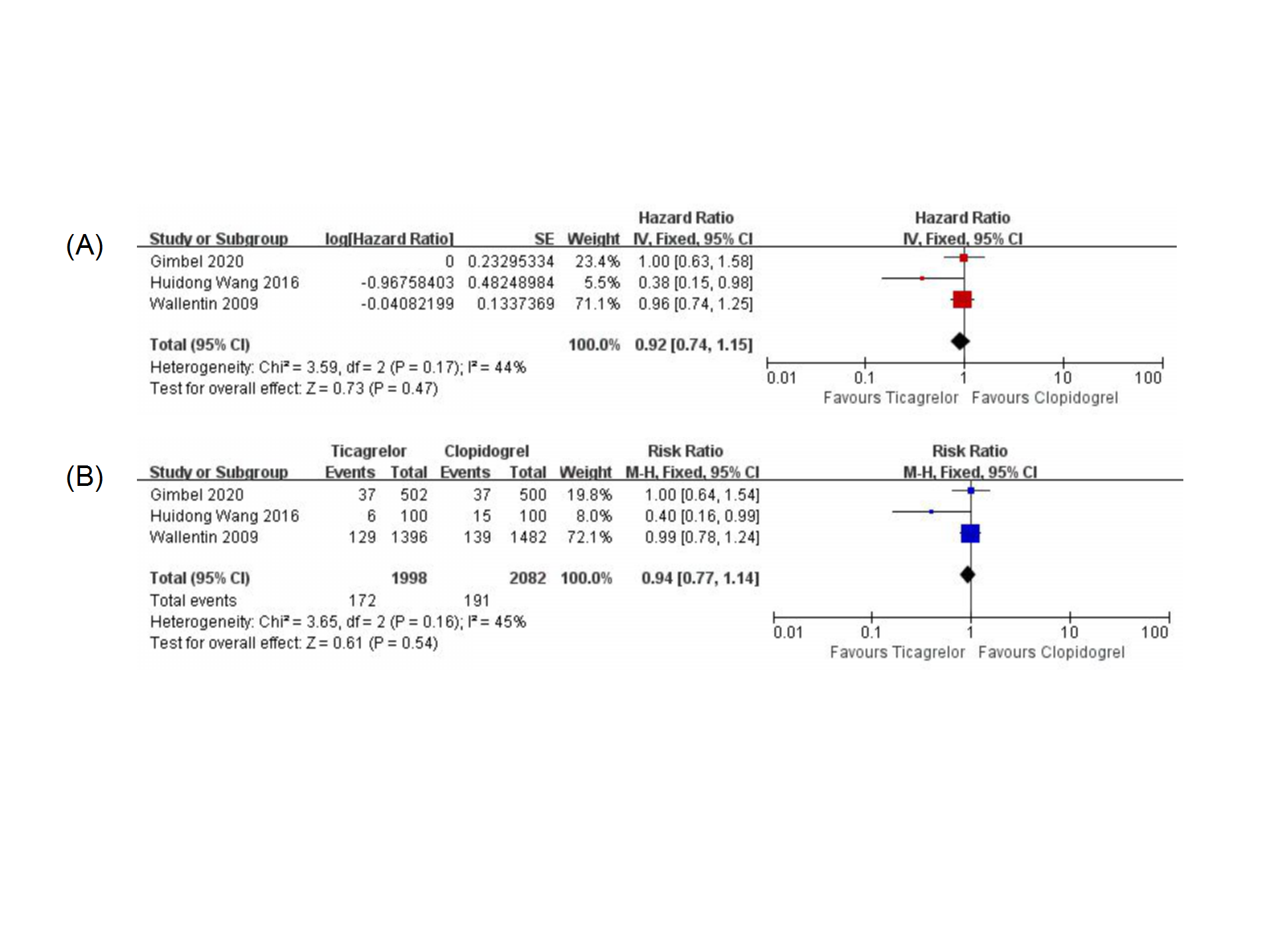

Supplement: Supplementary file 1 [file DataSheet1.ZIP › Supplementary files/Supplementary Figure 2.tif]

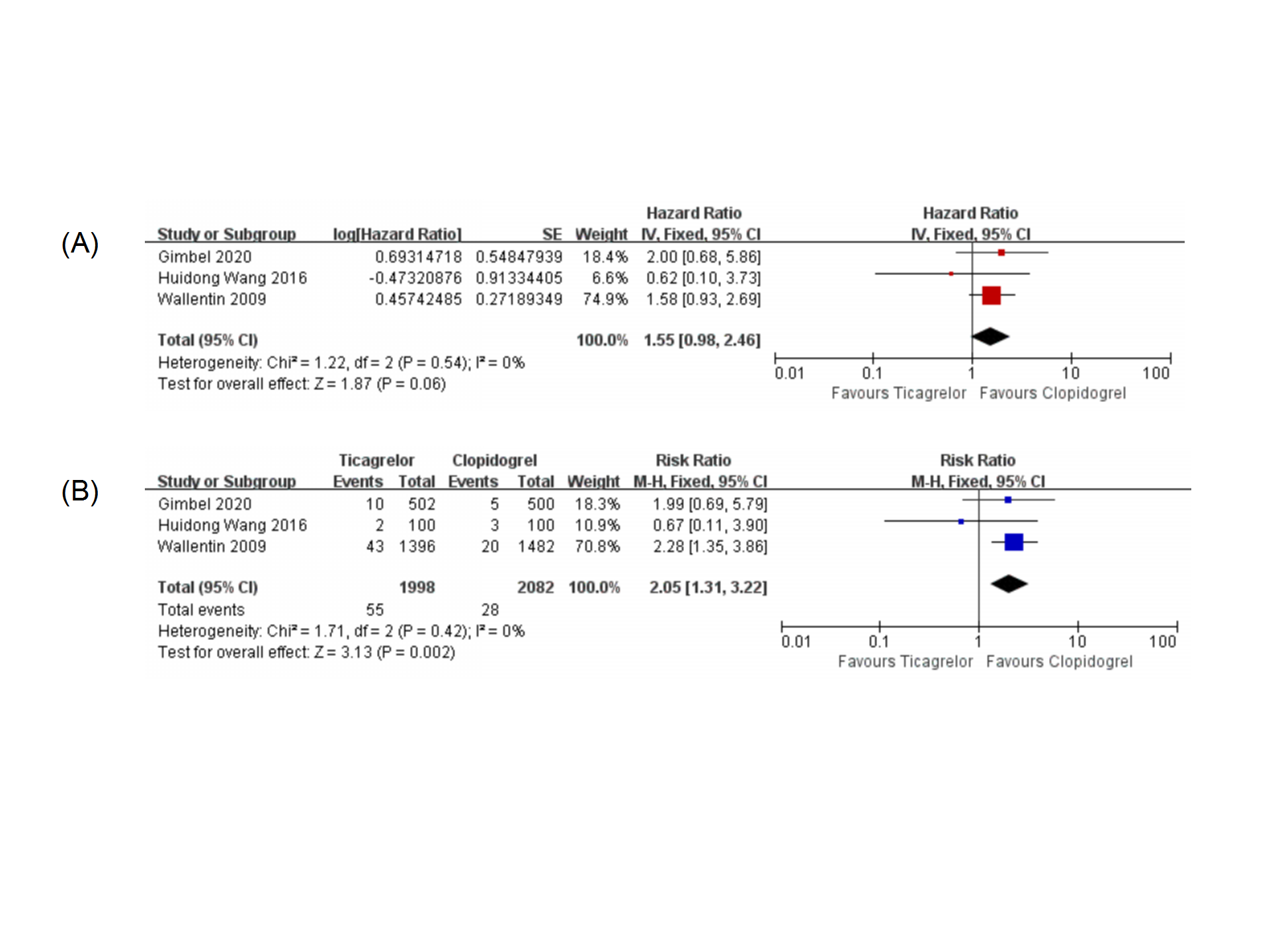

Supplement: Supplementary file 1 [file DataSheet1.ZIP › Supplementary files/Supplementary Figure 3.tif]

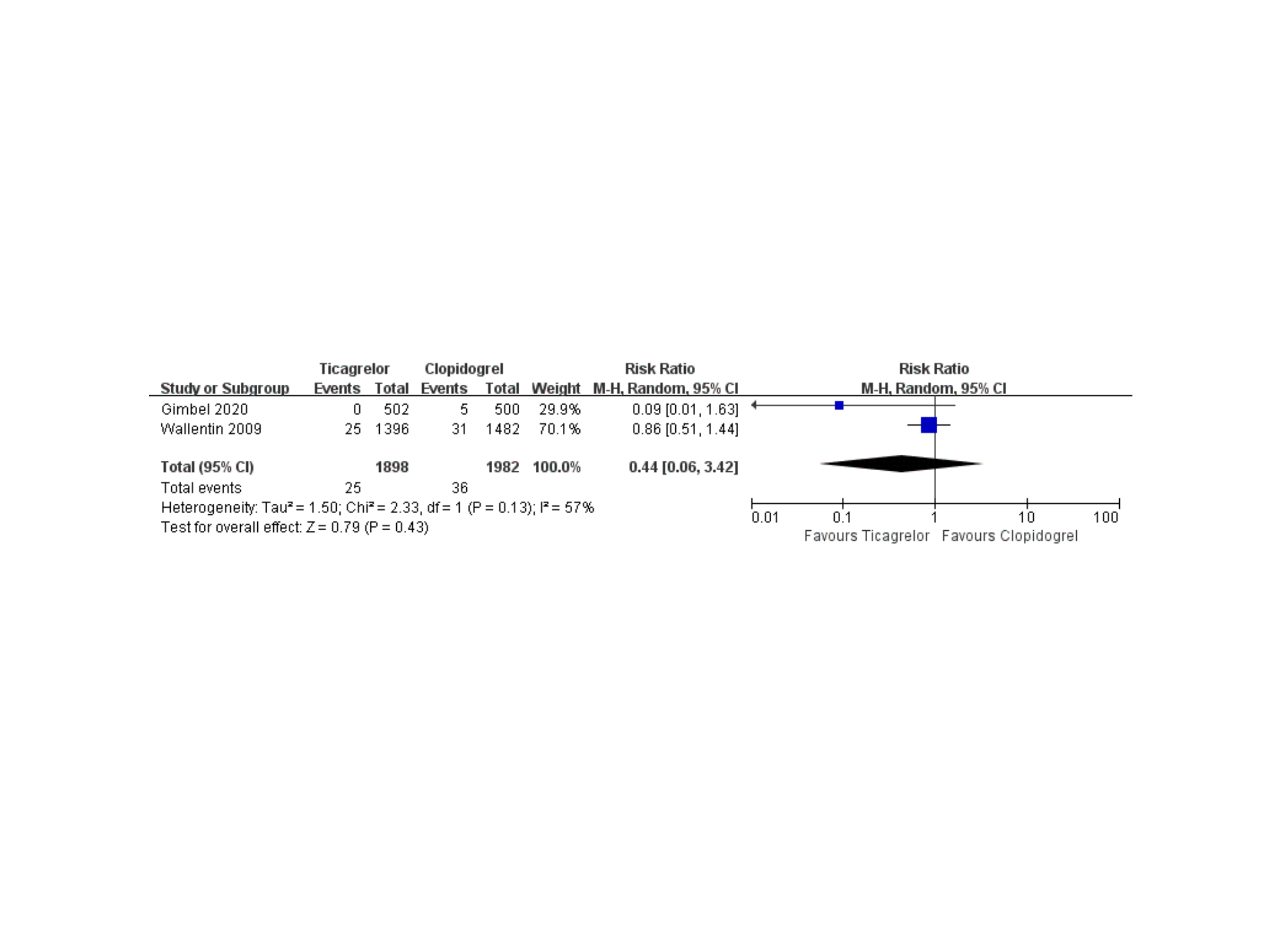

Supplement: Supplementary file 1 [file DataSheet1.ZIP › Supplementary files/Supplementary Figure 4.tif]

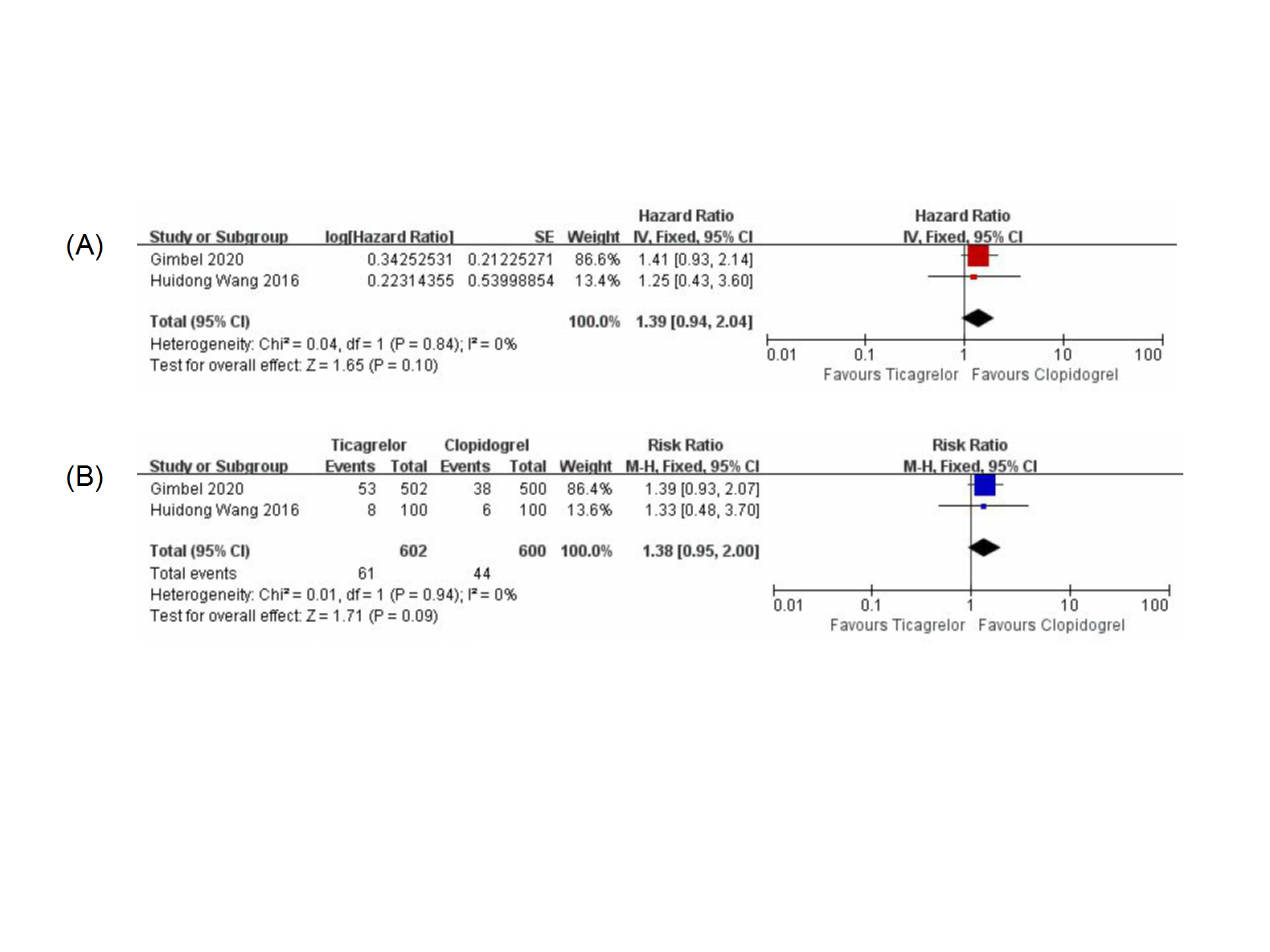

Supplement: Supplementary file 1 [file DataSheet1.ZIP › Supplementary files/Supplementary Figure 5.tif]

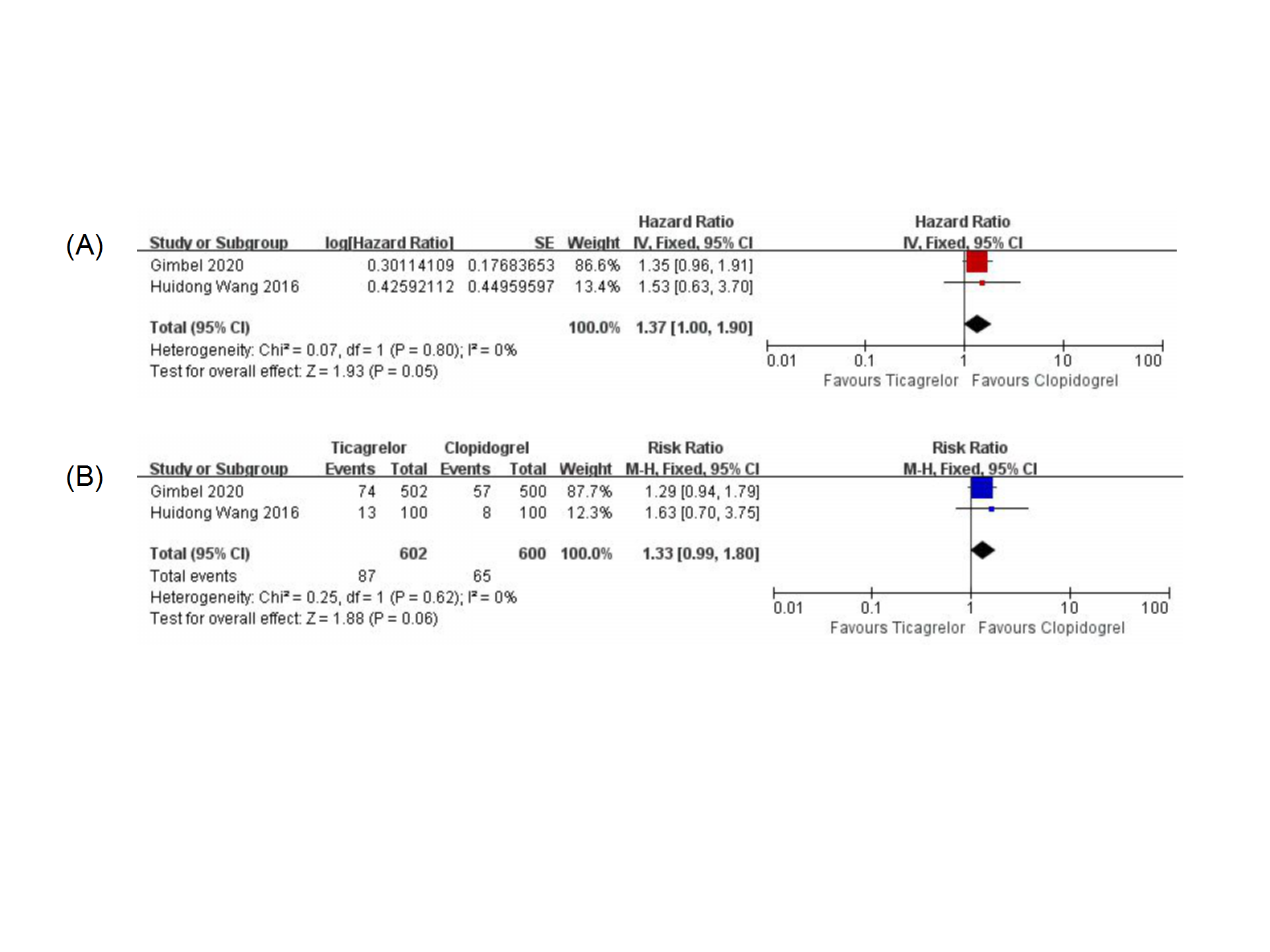

Supplement: Supplementary file 1 [file DataSheet1.ZIP › Supplementary files/Supplementary Figure 6.tif]
